# Supplementary material for: The impact of ESG performance on corporate innovation—Empirical evidence from Chinese pharmaceutical listed companies
Source: PLoS One. 2025 Nov 4;20(11):e0332906. doi: 10.1371/journal.pone.0332906 (PMC12585053; doi:10.1371/journal.pone.0332906)
Supplement: S1 File — Table 4. Correlation analysis of all variables. Table 5. Baseline regression results. Table 6. Results of robustness tests. Table 8. System GMM method. Table 9. Results of heterogeneity test for property attributes. Table 10. Results of the regional heterogeneity test. Table 11. Results of enterprise size heterogeneity test. Table 12 Mediation effects test based on R&D personnel. Table 13. Mediation effect tests based on government subsidies. (DOCX) [file pone.0332906.s001.DOCX]

**Table 4 Correlation analysis of all variables**

|  | RD | Patent | ESG | RDPeople | Sub | ROA | LEV | DEV | DUA | PID | CTR | CASH | ATO | TQ |
| --- | --- | --- | --- | --- | --- | --- | --- | --- | --- | --- | --- | --- | --- | --- |
| RD | 1 |  |  |  |  |  |  |  |  |  |  |  |  |  |
| Patent | 0.093*** | 1 |  |  |  |  |  |  |  |  |  |  |  |  |
| ESG | 0.257*** | 0.106*** | 1 |  |  |  |  |  |  |  |  |  |  |  |
| RDPeople | 0.798*** | 0.135*** | 0.229*** | 1 |  |  |  |  |  |  |  |  |  |  |
| Sub | 0.689*** | 0.106*** | 0.205*** | 0.613*** | 1 |  |  |  |  |  |  |  |  |  |
| ROA | 0.136*** | 0.047* | 0.304*** | 0.143*** | 0.081*** | 1 |  |  |  |  |  |  |  |  |
| LEV | 0.177*** | 0.042* | -0.239*** | 0.219*** | 0.290*** | -0.370*** | 1 |  |  |  |  |  |  |  |
| DEV | 0.091*** | 0.00800 | 0.0100 | 0.062** | 0.0150 | 0.296*** | -0.041* | 1 |  |  |  |  |  |  |
| DUA | -0.090*** | -0.060** | 0.00100 | -0.100*** | -0.145*** | 0.066*** | -0.063** | 0.063** | 1 |  |  |  |  |  |
| PID | -0.0390 | -0.0210 | 0.0330 | -0.082*** | -0.0100 | -0.0350 | 0.091*** | -0.0250 | 0.0410 | 1 |  |  |  |  |
| CTR | -0.056** | -0.00600 | -0.067*** | 0.0160 | 0.00400 | 0.0110 | 0.163*** | -0.054** | -0.073*** | 0.0150 | 1 |  |  |  |
| CASH | 0.153*** | 0.051** | 0.196*** | 0.152*** | 0.114*** | 0.630*** | -0.241*** | 0.177*** | 0.0260 | -0.044* | -0.054** | 1 |  |  |
| ATO | 0.194*** | 0.044* | 0.059** | 0.222*** | 0.200*** | 0.328*** | 0.181*** | 0.145*** | -0.106*** | -0.00700 | 0.106*** | 0.368*** | 1 |  |
| TQ | -0.050** | -0.0280 | 0.051** | -0.081*** | -0.126*** | 0.252*** | -0.187*** | 0.087*** | 0.075*** | -0.057** | -0.106*** | 0.180*** | -0.0100 | 1 |

Note: ***, **, and *, respectively, denote significance at the 1%, 5%, and 10% levels.

**Table 5 Baseline regression results.**

| **VARIABLES** | **(1)** | **(2)** | **(3)** | **(4)** | **(5)** | **(6)** |
| --- | --- | --- | --- | --- | --- | --- |
|  | **RD** | **RD** | **RD** | **Patent** | **Patent** | **Patent** |
| **ESG** | 0.248*** | 0.279*** | 0.220*** | 0.016** | 0.030*** | 0.021*** |
|  | (9.47) | (10.25) | (8.43) | (2.22) | (4.29) | (2.71) |
| **ROA** | 0.537 | 2.195*** | 1.323** | 0.166 | 0.108 | 0.149 |
|  | (0.95) | (3.63) | (2.39) | (1.38) | (1.01) | (1.22) |
| **LEV** | 1.862*** | 2.146*** | 1.797*** | 0.170*** | 0.157*** | 0.193*** |
|  | (8.38) | (10.32) | (8.27) | (2.87) | (3.28) | (3.25) |
| **DEV** | 0.145* | 0.166* | 0.151** | 0.010 | -0.005 | 0.006 |
|  | (1.87) | (1.91) | (2.03) | (0.62) | (-0.31) | (0.33) |
| **DUA** | -0.205*** | -0.219*** | -0.229*** | -0.023* | -0.033*** | -0.026* |
|  | (-3.72) | (-3.93) | (-4.31) | (-1.71) | (-2.72) | (-1.91) |
| **PID** | -0.668 | -1.739*** | -0.895 | -0.117 | -0.155 | -0.119 |
|  | (-1.14) | (-3.21) | (-1.51) | (-0.84) | (-1.36) | (-0.85) |
| **CTR** | -0.272*** | -0.311*** | -0.171** | -0.013 | -0.022 | -0.019 |
|  | (-3.47) | (-3.61) | (-2.17) | (-0.57) | (-0.95) | (-0.80) |
| **CASH** | 1.589*** | 0.883* | 0.760* | 0.111 | 0.164 | 0.136 |
|  | (3.48) | (1.73) | (1.70) | (0.81) | (1.26) | (1.00) |
| **ATO** | 0.423*** | 0.331** | 0.462*** | -0.050 | -0.020 | -0.063 |
|  | (2.67) | (2.32) | (2.95) | (-1.18) | (-0.58) | (-1.51) |
| **TQ** | -0.035** | -0.023 | -0.003 | -0.005 | -0.003 | -0.003 |
|  | (-2.00) | (-1.12) | (-0.19) | (-1.16) | (-0.95) | (-0.58) |
| **Constant** | 16.892*** | 17.056*** | 17.000*** | 0.033 | -0.013 | 0.012 |
|  | (68.99) | (74.68) | (69.67) | (0.52) | (-0.24) | (0.18) |
| **Observations** | 1,632 | 1,635 | 1,632 | 1,632 | 1,635 | 1,632 |
| **R-squared** | 0.544 | 0.219 | 0.586 | 0.145 | 0.034 | 0.154 |
| **CityCode FE** | YES |  | YES | YES |  | YES |
| **Year FE** |  | YES | YES |  | YES | YES |
| **F test** | 0 | 0 | 0 | 0.0372 | 2.57e-05 | 0.0166 |
| **F** | 30.91 | 37.72 | 28.13 | 1.932 | 3.934 | 2.181 |

Robust t-statistics in parentheses, *** p<0.01, ** p<0.05, * p<0.1

**Table 6 Results of robustness tests.**

| **VARIABLES** | **(1)** | **(2)** | **(3)** | **(4)** | **(5)** | **(6)** |
| --- | --- | --- | --- | --- | --- | --- |
|  | **RD 1** | **Patent 1** | **RD(q30)** | **RD(q60)** | **RD(q90)** | **Patent 2** |
| **ESG** |  |  | 0.194*** | 0.312*** | 0.408*** | 0.236** |
|  |  |  | (5.82) | (7.96) | (9.82) | (2.33) |
| **L.ESG** | 3.955*** | 0.014** |  |  |  |  |
|  | (7.34) | (1.97) |  |  |  |  |
| **ROA** | 37.828*** | -0.143 | 2.611*** | 1.645* | 1.035 | 2.211 |
|  | (3.54) | (-1.34) | (4.23) | (1.90) | (1.18) | (1.28) |
| **LEV** | 34.750*** | 0.171*** | 2.251*** | 2.239*** | 2.141*** | 2.517*** |
|  | (7.11) | (3.06) | (10.60) | (6.96) | (5.46) | (3.89) |
| **DEV** | 0.457 | 0.017 | 0.077 | 0.033 | 0.226 | 0.103 |
|  | (0.30) | (0.81) | (0.85) | (0.29) | (1.60) | (0.42) |
| **DUA** | -4.647*** | -0.031** | -0.063 | -0.102 | -0.454*** | -0.429** |
|  | (-3.91) | (-2.41) | (-0.85) | (-1.40) | (-4.34) | (-2.00) |
| **PID** | -22.421* | -0.059 | -0.835 | -1.245 | -2.310** | -1.264 |
|  | (-1.79) | (-0.41) | (-1.44) | (-1.60) | (-2.23) | (-0.66) |
| **CTR** | -2.447 | 0.005 | -0.292** | -0.373*** | -0.478*** | -0.249 |
|  | (-1.60) | (0.24) | (-2.03) | (-2.84) | (-3.74) | (-0.65) |
| **CASH** | 13.611 | 0.295** | 1.395** | 1.989*** | 0.197 | 1.710 |
|  | (1.41) | (2.46) | (2.45) | (3.01) | (0.19) | (1.01) |
| **ATO** | 14.375*** | -0.023 | 0.569*** | 0.147 | -0.100 | -0.786 |
|  | (4.29) | (-0.53) | (3.60) | (0.73) | (-0.34) | (-1.51) |
| **TQ** | -0.215 | 0.015*** | -0.060** | -0.044 | 0.027 | -0.045 |
|  | (-0.50) | (2.72) | (-2.15) | (-1.51) | (0.59) | (-0.78) |
| **Constant** | 77.405*** | -0.053 | 16.301*** | 17.064*** | 18.526*** | -4.582*** |
|  | (14.93) | (-0.80) | (57.53) | (49.51) | (42.56) | (-4.49) |
| **Observations** | 1,372 | 1,372 | 1,635 | 1,635 | 1,635 | 1,635 |
| **R-squared** | 0.556 | 0.212 |  |  |  |  |
| **CityCode FE** | YES | YES |  |  |  | YES |
| **Year FE** | YES | YES |  |  |  | YES |
| **F test** | 0 | 0.00236 |  |  |  | 0 |
| **F** | 26.30 | 2.748 |  |  |  | . |

Robust t-statistics in parentheses, *** p<0.01, ** p<0.05, * p<0.1

**Table 8 System GMM method.**

| **VARIABLES** | **(1)** | **(2)** |
| --- | --- | --- |
|  | **RD** | **Patent** |
| **L.RD** | 0.973***(0.029) |  |
| **ESG** | 0.104**(0.049) | 0.245**(0.119) |
| **L.Patent** |  | -0.519***(0.076) |
| **ROA** | 0.040(0.538) | -3.372(2.222) |
| **LEV** | -0.126(0.167) | 0.531(0.917) |
| **DEV** | 0.330***(0.047) | -0.395(0.282) |
| **DUA** | -0.037(0.073) | 0.059(0.166) |
| **PID** | -1.564*(0.854) | -1.497(1.789) |
| **CTR** | 0.033(0.146) | 0.275(0.299) |
| **CASH** | -0.265(0.245) | -0.936(1.006) |
| **ATO** | 0.124(0.150) | 1.431**(0.724) |
| **TQ** | -0.016(0.011) | -0.134**(0.061) |
| **Constant** | 0.749 | -1.090 |
|  | (0.665) | (1.108) |
| **Number of id** | 248 | 248 |
| **AR（1）** | -5.940 | -2.967 |
| **AR（1）p** | 2.85e-09 | 0.00301 |
| **AR（2）** | -0.414 | -1.465 |
| **AR（2）p** | 0.679 | 0.143 |
| **Hansen** | 45.04 | 36.42 |
| **Hansenp** | 0.171 | 0.195 |
| **N** | 1375 | 1375 |

Standard errors in parentheses, *** p<0.01, ** p<0.05, * p<0.1

**Table 9 Results of heterogeneity test for property attributes.**

| **VARIABLES** | **（1）** | **（2）** | **（3）** | **(4)** |
| --- | --- | --- | --- | --- |
|  | **non-SOEs** | **SOEs** | **non-SOEs** | **SOEs** |
|  | **RD** | **RD** | **Patent** | **Patent** |
| **ESG** | 0.195*** | 0.181*** | 0.019** | 0.010 |
|  | [6.46] | [3.97] | [2.19] | [0.51] |
| **ROA** | 1.385** | 2.143** | 0.232* | -0.561 |
|  | [2.25] | [2.36] | [1.75] | [-1.56] |
| **LEV** | 1.568*** | 2.759*** | 0.179*** | 0.072 |
|  | [5.81] | [6.47] | [2.62] | [0.55] |
| **DEV** | 0.149* | -0.064 | 0.004 | 0.048 |
|  | [1.93] | [-0.34] | [0.21] | [1.18] |
| **DUA** | -0.309*** | 0.035 | -0.032** | -0.033 |
|  | [-4.99] | [0.34] | [-2.14] | [-0.75] |
| **PID** | -0.964 | -0.633 | -0.075 | -0.069 |
|  | [-1.27] | [-0.77] | [-0.44] | [-0.22] |
| **CTR** | -0.132 | -0.060 | -0.014 | -0.021 |
|  | [-1.27] | [-0.49] | [-0.48] | [-0.49] |
| **CASH** | 0.006 | 1.895*** | 0.147 | -0.106 |
|  | [0.01] | [3.03] | [0.87] | [-0.41] |
| **ATO** | 0.528*** | 0.538** | -0.120** | 0.039 |
|  | [2.64] | [2.12] | [-2.50] | [0.53] |
| **TQ** | 0.008 | -0.145*** | -0.003 | 0.004 |
|  | [0.42] | [-3.70] | [-0.66] | [0.37] |
| **Constant** | 17.203*** | 16.852*** | 0.031 | 0.068 |
|  | [56.35] | [49.04] | [0.40] | [0.43] |
| **N** | 1297 | 331 | 1297 | 331 |
| **CityCode FE** | YES | YES | YES | YES |
| **Year FE** | YES | YES | YES | YES |
| **r2_a** | 0.524 | 0.788 | 0.072 | 0.171 |
| **F** | 17.368 | 11.212 | 1.871 | 0.648 |

t statistics in brackets, * p < 0.1, ** p < 0.05, *** p < 0.01

**Table 10 Results of the regional heterogeneity test.**

| **VARIABLES** | **（1）** | **（2）** | **（3）** | **(4)** |
| --- | --- | --- | --- | --- |
|  | **Midwest** | **East** | **Midwest** | **East** |
|  | **RD** | **RD** | **Patent** | **Patent** |
| **ESG** | 0.178*** | 0.234*** | 0.013 | 0.025*** |
|  | [4.64] | [6.91] | [0.89] | [2.72] |
| **ROA** | 2.179*** | 1.094 | 0.320 | 0.076 |
|  | [2.64] | [1.54] | [1.46] | [0.51] |
| **LEV** | 1.691*** | 1.878*** | 0.288*** | 0.145** |
|  | [4.81] | [6.98] | [2.65] | [2.02] |
| **DEV** | 0.014 | 0.237** | -0.005 | 0.019 |
|  | [0.14] | [2.46] | [-0.22] | [0.83] |
| **DUA** | -0.308*** | -0.174*** | -0.047** | -0.009 |
|  | [-3.43] | [-2.64] | [-2.08] | [-0.54] |
| **PID** | 0.828 | -1.734** | 0.052 | -0.221 |
|  | [0.94] | [-2.33] | [0.22] | [-1.26] |
| **CTR** | -0.151 | -0.151 | -0.033 | -0.004 |
|  | [-1.38] | [-1.39] | [-0.76] | [-0.12] |
| **CASH** | 2.206*** | -0.435 | 0.235 | 0.051 |
|  | [3.35] | [-0.74] | [1.23] | [0.26] |
| **ATO** | -0.429* | 1.014*** | -0.204*** | 0.017 |
|  | [-1.70] | [5.41] | [-3.00] | [0.33] |
| **TQ** | 0.008 | -0.003 | -0.000 | -0.003 |
|  | [0.29] | [-0.12] | [-0.02] | [-0.56] |
| **Constant** | 16.519*** | 17.259*** | 0.014 | 0.006 |
|  | [45.88] | [53.71] | [0.13] | [0.07] |
| **N** | 678 | 954 | 678 | 954 |
| **CityCode FE** | YES | YES | YES | YES |
| **Year FE** | YES | YES | YES | YES |
| **r2_a** | 0.593 | 0.485 | 0.083 | 0.084 |
| **F** | 9.536 | 27.870 | 1.523 | 1.602 |

t statistics in brackets, * p < 0.1, ** p < 0.05, *** p < 0.01

**Table 11 Results of enterprise size heterogeneity test.**

| **VARIABLES** | **（1）** | **（2）** | **（3）** | **(4)** |
| --- | --- | --- | --- | --- |
|  | **SMEs** | **LEs** | **SMEs** | **LEs** |
|  | **RD** | **RD** | **Patent** | **Patent** |
| **ESG** | 0.097*** | 0.168*** | 0.015* | 0.002 |
|  | [3.66] | [5.06] | [1.76] | [0.12] |
| **ROA** | 0.153 | 0.886 | 0.157 | 0.292 |
|  | [0.26] | [1.39] | [1.25] | [1.02] |
| **LEV** | 0.442** | 1.476*** | 0.069 | 0.384*** |
|  | [2.01] | [4.58] | [1.09] | [2.74] |
| **DEV** | 0.195** | -0.021 | 0.011 | -0.006 |
|  | [2.40] | [-0.28] | [0.45] | [-0.28] |
| **DUA** | -0.040 | 0.009 | -0.036** | 0.035 |
|  | [-0.68] | [0.11] | [-2.37] | [0.89] |
| **PID** | 0.138 | -1.902*** | -0.165 | 0.485 |
|  | [0.21] | [-2.69] | [-1.02] | [1.46] |
| **CTR** | -0.165* | -0.250** | -0.043** | 0.016 |
|  | [-1.90] | [-2.55] | [-1.98] | [0.22] |
| **CASH** | 0.248 | 0.133 | -0.048 | 0.102 |
|  | [0.46] | [0.23] | [-0.29] | [0.43] |
| **ATO** | -0.269 | -0.650*** | 0.013 | -0.218** |
|  | [-1.40] | [-2.97] | [0.25] | [-2.05] |
| **TQ** | 0.034* | 0.057** | -0.001 | -0.005 |
|  | [1.79] | [2.16] | [-0.26] | [-0.37] |
| **Constant** | 17.356*** | 19.158*** | 0.060 | -0.101 |
|  | [65.28] | [57.18] | [0.82] | [-0.62] |
| **N** | 1136 | 484 | 1136 | 484 |
| **CityCode FE** | YES | YES | YES | YES |
| **Year FE** | YES | YES | YES | YES |
| **r2_a** | 0.499 | 0.697 | 0.095 | 0.186 |
| **F** | 3.410 | 7.622 | 1.563 | 1.253 |

t statistics in brackets, * p < 0.1, ** p < 0.05, *** p < 0.01

**Table 12 Mediation effects test based on R&D personnel.**

| **VARIABLES** | **(1)** | **(2)** | **(3)** | **(4)** | **(5)** | **(6)** |
| --- | --- | --- | --- | --- | --- | --- |
|  | **RD** | **RDPeople** | **RD** | **Patent** | **RDPeople** | **Patent** |
| **ESG** | 0.220*** | 0.167*** | 0.077*** | 0.021*** | 0.167*** | 0.016** |
|  | (8.43) | (8.18) | (4.37) | (2.71) | (8.18) | (2.10) |
| **RDPeople** |  |  | 0.858*** |  |  | 0.028** |
|  |  |  | (32.24) |  |  | (2.40) |
| **ROA** | 1.323** | 1.343*** | 0.170 | 0.149 | 1.343*** | 0.111 |
|  | (2.39) | (3.23) | (0.44) | (1.22) | (3.23) | (0.92) |
| **LEV** | 1.797*** | 1.462*** | 0.542*** | 0.193*** | 1.462*** | 0.152*** |
|  | (8.27) | (8.42) | (3.92) | (3.25) | (8.42) | (2.63) |
| **DEV** | 0.151** | -0.010 | 0.160*** | 0.006 | -0.010 | 0.006 |
|  | (2.03) | (-0.20) | (2.82) | (0.33) | (-0.20) | (0.35) |
| **DUA** | -0.229*** | -0.149*** | -0.101** | -0.026* | -0.149*** | -0.022 |
|  | (-4.31) | (-3.36) | (-2.53) | (-1.91) | (-3.36) | (-1.61) |
| **PID** | -0.895 | -1.196** | 0.132 | -0.119 | -1.196** | -0.085 |
|  | (-1.51) | (-2.53) | (0.33) | (-0.85) | (-2.53) | (-0.61) |
| **CTR** | -0.171** | -0.089 | -0.095 | -0.019 | -0.089 | -0.016 |
|  | (-2.17) | (-1.39) | (-1.52) | (-0.80) | (-1.39) | (-0.69) |
| **CASH** | 0.760* | 0.558 | 0.282 | 0.136 | 0.558 | 0.120 |
|  | (1.70) | (1.46) | (0.84) | (1.00) | (1.46) | (0.89) |
| **ATO** | 0.462*** | 0.489*** | 0.041 | -0.063 | 0.489*** | -0.076* |
|  | (2.95) | (4.04) | (0.38) | (-1.51) | (4.04) | (-1.83) |
| **TQ** | -0.003 | -0.022 | 0.015 | -0.003 | -0.022 | -0.002 |
|  | (-0.19) | (-1.41) | (1.28) | (-0.58) | (-1.41) | (-0.44) |
| **Constant** | 17.000*** | 4.572*** | 13.075*** | 0.012 | 4.572*** | -0.117 |
|  | (69.67) | (22.73) | (66.94) | (0.18) | (22.73) | (-1.36) |
| **Observations** | 1,632 | 1,632 | 1,632 | 1,632 | 1,632 | 1,632 |
| **R-squared** | 0.586 | 0.535 | 0.796 | 0.154 | 0.535 | 0.159 |
| **F test** | 0 | 0 | 0 | 0.0166 | 0 | 0.0154 |
| **r2_a** | 0.551 | 0.497 | 0.779 | 0.0842 | 0.497 | 0.0887 |
| **F** | 28.13 | 29.32 | 160.4 | 2.181 | 29.32 | 2.139 |
| **Sobel Test** | 0.1433***（z= 8.635） | | | 0.0047***（z= 2.764） | | |
| **Goodman Test1** | 0.1433***（z= 8.633） | | | 0.0047***（z= 2.748） | | |
| **Goodman Test2** | 0.1433***（z= 8.638） | | | 0.0047***（z= 2.780） | | |
| **Indirect effect** | 0.1433***（z= 8.635） | | | 0.0047***（z= 2.764） | | |
| **Direct effect** | 0.0766***（z= 4.685） | | | 0.0162**（z= 2. 237） | | |
| **Total effect** | 0.2199***（z= 9.680） | | | 0.0209***（z= 2. 951） | | |
| **Indirect effect to total effect ratio** | 0.6518 | | | 0. 2243 | | |
| **Bootstrap Method Indirect Effects Test** | 0.1433***（LLCI=0.1403, ULCI=0.1439） | | | 0.0047***（LLCI=0.0037, ULCI=0.0059） | | |
|  | Effective mechanisms-Positive transfer | | | Effective mechanisms-Positive transfer | | |

Robust t-statistics in parentheses, *** p<0.01, ** p<0.05, * p<0.1

**Table 13 Mediation effect tests based on government subsidies.**

| **VARIABLES** | **(1)** | **(2)** | **(3)** | **(4)** | **(5)** | **(6)** |
| --- | --- | --- | --- | --- | --- | --- |
|  | **RD** | **Sub** | **RD** | **Patent** | **Sub** | **Patent** |
| **ESG** | 0.220*** | 0.224*** | 0.102*** | 0.021*** | 0.224*** | 0.016** |
|  | (8.43) | (8.00) | (4.97) | (2.71) | (8.00) | (2.10) |
| **Sub** |  |  | 0.524*** |  |  | 0.020*** |
|  |  |  | (23.49) |  |  | (2.64) |
| **ROA** | 1.323** | 1.798*** | 0.380 | 0.149 | 1.798*** | 0.112 |
|  | (2.39) | (3.53) | (0.83) | (1.22) | (3.53) | (0.93) |
| **LEV** | 1.797*** | 2.507*** | 0.483*** | 0.193*** | 2.507*** | 0.142** |
|  | (8.27) | (12.18) | (2.78) | (3.25) | (12.18) | (2.46) |
| **DEV** | 0.151** | -0.101 | 0.204*** | 0.006 | -0.101 | 0.008 |
|  | (2.03) | (-1.37) | (3.48) | (0.33) | (-1.37) | (0.47) |
| **DUA** | -0.229*** | -0.320*** | -0.061 | -0.026* | -0.320*** | -0.019 |
|  | (-4.31) | (-5.41) | (-1.39) | (-1.91) | (-5.41) | (-1.44) |
| **PID** | -0.895 | -2.174*** | 0.244 | -0.119 | -2.174*** | -0.074 |
|  | (-1.51) | (-3.49) | (0.52) | (-0.85) | (-3.49) | (-0.54) |
| **CTR** | -0.171** | -0.080 | -0.129** | -0.019 | -0.080 | -0.017 |
|  | (-2.17) | (-0.92) | (-2.26) | (-0.80) | (-0.92) | (-0.73) |
| **CASH** | 0.760* | 1.480*** | -0.015 | 0.136 | 1.480*** | 0.105 |
|  | (1.70) | (2.92) | (-0.04) | (1.00) | (2.92) | (0.77) |
| **ATO** | 0.462*** | 0.323** | 0.292** | -0.063 | 0.323** | -0.069* |
|  | (2.95) | (1.98) | (2.25) | (-1.51) | (1.98) | (-1.66) |
| **TQ** | -0.003 | -0.061*** | 0.029** | -0.003 | -0.061*** | -0.001 |
|  | (-0.19) | (-3.07) | (2.10) | (-0.58) | (-3.07) | (-0.29) |
| **Constant** | 17.000*** | 15.731*** | 8.755*** | 0.012 | 15.731*** | -0.310** |
|  | (69.67) | (59.00) | (21.99) | (0.18) | (59.00) | (-2.31) |
| **Observations** | 1,632 | 1,632 | 1,632 | 1,632 | 1,632 | 1,632 |
| **R-squared** | 0.586 | 0.523 | 0.730 | 0.154 | 0.523 | 0.158 |
| **F test** | 0 | 0 | 0 | 0.0166 | 0 | 0.0120 |
| **r2_a** | 0.551 | 0.484 | 0.708 | 0.0842 | 0.484 | 0.0886 |
| **F** | 28.13 | 35.13 | 90.14 | 2.181 | 35.13 | 2.210 |
| **Sobel Test** | 0.1175***（z= 8.367） | | | 0.0046***（z= 2.735） | | |
| **Goodman Test1** | 0.1175***（z= 8.363） | | | 0.0046***（z= 2.719） | | |
| **Goodman Test2** | 0.1175***（z= 8.372） | | | 0.0046***（z= 2.751） | | |
| **Indirect effect** | 0.1175***（z= 8.367） | | | 0.0046***（z= 2.735） | | |
| **Direct effect** | 0.1024***（z= 5.447） | | | 0.0163**（z= 2.252） | | |
| **Total effect** | 0.2199***（z= 9.680） | | | 0.0209***（z= 2.951） | | |
| **Indirect effect to total effect ratio** | 0. 5344 | | | 0. 2195 | | |
| **Bootstrap Method Indirect Effects Test** | 0.1175***（LLCI=0.1076, ULCI=0.1435） | | | 0.0046***（LLCI=0.0029, ULCI=0.0058） | | |
|  | Effective mechanisms-Positive transfer | | | Effective mechanisms-Positive transfer | | |

Robust t-statistics in parentheses, *** p<0.01, ** p<0.05, * p<0.1
