# Supplementary material for: The impact of ESG performance on corporate innovation—Empirical evidence from Chinese pharmaceutical listed companies
Source: PLoS One. 2025 Nov 4;20(11):e0332906. doi: 10.1371/journal.pone.0332906 (PMC12585053; doi:10.1371/journal.pone.0332906)
Supplement: Attachment — Submitted filename: Response to Reviewers.docx [file pone.0332906.s003.docx]

**Response to Reviewers**

**Response Reviewer 1**

We are very grateful to the reviewers for their review and valuable comments on our papers. Your suggestions are crucial to improve the quality of the paper. We have made changes according to your comments and our response is as follows:

**Commentator's Opinion:**

**a: Introduction：The section lacks an outline of the article's marginal contributions and an overview of each section.**

**Author's Response:**

We agree with this view and have added an outline of the marginal contribution of the article and a summary of each section to the introduction of the article. See page 5 for specific changes.

**Commentator's Opinion:**

**b1: It is recommended that the background discussion should be extensive and the hypothesis development should be thoroughly examined through an extensive literature review. It is suggested to include references on government subsidies, such as: Han, Y., Han, L., Liu, C., & Wang, Q. (2024). How does government R&D subsidies affect enterprises’ viability? An investigation on inverted U-shaped relationship[J]. Finance Research Letters, 106235.** **<https://doi.org/10.1016/j.frl.2024.106235>**

**Author's Response:**

The theoretical foundations section has been reorganised with relevant paragraphs based on your suggestions, and references to government subsidies (https://doi.org/10.1016/j.frl.2024.106235) have also been added, see the Theoretical Analysis and Research Hypotheses section on pages 6-11 for specific changes.

**Commentator's Opinion:**

**b2: I did not see an in-depth analysis of the Theoretical Basis. It is recommended to add Institutional background.**

**Author's Response:**

We agree with your suggestion for an in-depth analysis of the theoretical underpinnings, and we have reworked the Theoretical Analysis and Research Assumptions section in accordance with your suggestion, which can be found in the Theoretical Analysis and Research Assumptions section on pages 6-11. We believe the revised version is even better.

**Commentator's Opinion:**

**c1: Please explain proprietary names such as ST, *ST, and PT to enable the reader to better understand their specific meaning, e.g. ST companies (Companies with special treatment due to two consecutive years of operating losses).**

**Author's Response:**

We agree with this view and we have added an explanation of the proprietary name, as modified on page 12, line 238.

**Commentator's Opinion:**

**c2: The authors selected China's A-share pharmaceutical listed enterprises from 2015 to 2022 as the research sample. It would be beneficial if the authors could explain further and justify the strong reasons for only using pharmaceutical listed enterprises from 2015 to 2022. Is there any possibility of bias? If so, how will the authors address this issue?**

**Author's Response:**

We agree with this view. Firstly, the relationship between sustainable development and corporate innovation is one of the key concerns of pharmaceutical companies, so the main research target of this paper is pharmaceutical companies. Secondly, the rationale for using data from 2015 - 2022 has been added, as described in the Sample Selection and Data Sources section on pages 11-12. Finally, the article limitations have been added at the end of the article, with the opportunity to further explore other industries as research samples in the future, as shown on pages 44-45.

**Commentator's Opinion:**

**d1: The choice of instrumental variables in the endogeneity discussion is not appropriate, and the results are not robust enough.**

**Author's Response:**

We thank the reviewers for their suggestions regarding the endogeneity discussion. New references have been added, and the analysis of the KP-LM statistic shows that the probability of under-identification of the instrumental variables is significant and zero, which strongly rejects the original hypothesis of ‘unidentifiable’, confirms the correlation between the instrumental variables and the core explanatory variables, and shows that the instrumental variables selected in this paper are valid and reliable. See the discussion of endogeneity on page 23.

**Commentator's Opinion:**

**d2: Although the article conducts mechanism examinations and heterogeneity analyses, these sections are too brief.**

**Author's Response:**

We thank the reviewers for their suggestions to add content to the chapters on mechanism testing and heterogeneity analysis. Due to space constraints, we are unable to discuss this in detail in this paper. We plan to explore this issue further in future studies.

**Response Reviewer 2**

We are very grateful to the reviewers for their review and valuable comments on our papers. Your suggestions are crucial to improve the quality of the paper. We have made changes according to your comments and our response is as follows:

**Commentator's Opinion:**

**1) Both ESG and enterprise innovation are latent variables, that is, it is not advisable to measure them by a single value. Thus, the appropriate statistical technique to understand the relationships between the variables and test the hypotheses would be through structural equation modeling and not regression. If a regression were to be used, it should be of the "Ordinal Regression" type, which would allow it to model the dependence of a polytomous ordinal response on a set of predictors, which can be factors or covariates. The Ordinal Regression design is based on the methodology of McCullagh (1980, 1998). This is because the ESG variable was collected in such a way as to be presented as an ordinary variable.**

**Author's Response:**

We thank the reviewers for their suggestion regarding regression. However, due to limitations in ESG-related data collection, we are unable to implement this suggestion for the time being, while referring to the methodology of related studies (<https://doi.org/10.1371/journal.pone.0301701>;

https://doi.org/10.1371/journal. pone.0310447), we chose to keep the current methodology, which is related on pp. 14-15. We will attempt to address this issue in future research.

**Commentator's Opinion:**

**2) Saying that ESG can be measured by "According to CSI ESG rating from low to high, the value is 1~9" is a very simplistic approach. In many studies, ESG is made up of more than a dozen variables. It is much more advisable for ESG to be a latent variable than an observable variable.**

**Author's Response:**

We thank the reviewers for their suggestion regarding the measurement of ESG variables. Due to limitations in ESG-related data collection, we are unable to implement this suggestion at this time. In addition, we have added the addition of using this method to measure ESG in the Sample Selection and Data Sources section and in the Selection of Variables for ESG section, as modified on page 14, line 293. We will attempt to address this issue in future research.

**Commentator's Opinion:**

**3) Converting ordinal variables into numerical variables is not appropriate. It is a statistically incorrect conversion and can generate distorted or unrealistic values. I understand that in PLOSONE alone there have been more than 16 articles in the last two years using this information incorrectly (https://doi.org/10.1371/journal.pone.0310447; https://doi.org/10.1371/journal.pone.0299184; https://doi.org/10.1371/journal.pone.0307535; https://doi.org/10.1371/journal.pone.0295548; https://doi.org/10.1371/journal.pone.0313686; https://doi.org/10.1371/journal.pone.0301701, just to name a few). As an reviewer, I cannot help but point out that the conversion of ordinal variables into numeric variables and the statistical technique (regression for latent variables) used are incorrect and cannot be done in this way. The result generated ends up not having statistical validity. The correct thing to do would be to collect the variables in quantitative format and use structural equation modeling.**

**Author's Response:**

We thank the reviewers for their suggestions regarding variable conversion. We have added citations of relevant literature using this method to measure ESG in the Variable Selection and Setting for ESG section as per your suggestion to use the article published in PLos one on variable measurement for ESG, see page 14, line 297 for the specific modification. We plan to make further attempts to address this issue in future studies.

**Response Reviewer 3**

We are very grateful to the reviewers for their review and valuable comments on our papers. Your suggestions are crucial to improve the quality of the paper. We have made changes according to your comments and our response is as follows:

**Commentator's Opinion:**

**1. The abstract should be improved. Your findings can be expressed more clearly in the abstract, which should also briefly mention the policy implications.**

**Author's Response:**

We agree with this view and have rephrased the findings in the summary section and added references to policy implications. See page 2 for specific changes.

**Commentator's Opinion:**

**2. The study's aim and contribution should be expressed more strongly. The introduction needs to be more relevant to the discussion. The introduction's discussion and research contributions should be redesigned.**

**Author's Response:**

We have reorganized the introductory and discussion-related paragraphs and believe that the revised version is even better. See pages 3-5 and 43-45 for specific changes.

**Commentator's Opinion:**

**3. There needs to be more literature review and discussion of why this topic matters and how it fills a gap in the literature.**

**Author's Response:**

We agree with this view and have added a further collation of the ESG related literature review in the introduction section. In addition, a discussion of the importance of green innovation for firms has been added. Finally, the marginal contribution of the article has been added to provide further discussion on filling gaps in the literature. See pages 3-5 for specific revisions.

**Commentator's Opinion:**

**4. Control variables, such as PID (percentage of independent directors) and DUA (second job alignment), are typically used in corporate governance studies. They may need more relevance to the specific innovation outcomes being modeled.**

**Author's Response:**

We thank the reviewers for raising the issue that individual control variables may need to be more relevant to the particular innovation being modelled. However, based on existing studies in the literature demonstrating the correlation of PID and DUA with firm innovation (https://doi.org/10.3390/ijerph19148558). Therefore, we retain the current choice of control variables. The details are as follows:

Two duty unification（DUA）: If the chairman and CEO are held by the same person, if the chairman and CEO are held by the same person, the corporate governance structure will be weakened, and the enterprise decision-making will lack democracy, which will have an adverse impact on green R&D.

Proportion of independent directors（PID）: When the proportion of independent directors is high, it indicates that the board of directors has strong independence, which can promote the improvement of enterprise performance and have a positive impact on green innovation.

**Commentator's Opinion:**

**5. Some control variables, such as ROA, TQ, and LEV, might be highly correlated with each other, which could affect the stability of coefficient estimates in regression models.**

**Author's Response:**

We thank the reviewers for raising the issue that some control variables can affect the stability of coefficient estimates in regression models. First, based on the existing literature (<https://doi.org/10.3390/ijerph19148558>;

<https://doi.org/10.3389/fpsyg.2023.1096419>;

Li Q, Xiao Z. Heterogeneous Environmental Regulation Tools and Green Innovation Incentives: Evidence from Green Patents of Listed Companies. Economic Research Journal. (2020)55: 192-208.) These control variables have a solid theoretical foundation; Second, we calculated the matrix of correlation coefficients between all variables (see S1 Table for details) and found that certain variables were indeed moderately correlated (with correlation coefficients between 0.4 and 0.6), but not to the extent of being highly correlated (>0.8); Finally, we further calculated the variance inflation factor (VIF), and the VIF values for all variables were less than 5, indicating that the multicollinearity problem was not serious. Therefore, we chose to keep these current control variables.

**Commentator's Opinion:**

**6. There are typo mistakes and format issues. Such as line 212: “innovation inpu” and line 216: Error! Reference source not found.**

**Author's Response:**

We agree with these suggestions of yours and have rechecked and corrected the typos and formatting issues.

**Response Reviewer 4**

**Commentator's Opinion:**

**I suggest synthesizing the abstract, removing elements of detail, reviewing it following the IMRaD (Introduction-Methods-Results-Discussion) model, frequently used in empirical research in the natural and social sciences. In particular, the reference is to Methods M.**

**Author's Response:**

We agree with you on this point and the abstract section has been reworked and a section on the methodological presentation of the article has been added. See page 2 for specific revisions.

**Commentator's Opinion:**

**I would suggest presenting the objective now and better supporting the rest of the content, For example, some quotes could be added to support this: "The comprehensive advancement of China's manufacturing sector continues to demonstrate elevated energy consumption and significant pollution, accompanied by a low resource utilization rate resulting in the degradation of the ecological environment.".**

**Other suggestions for integration: formalize the research question and highlight the classic "Section' summary passage that closes introductory manuscripts.**

**Author's Response:**

We agree with these suggestions of yours and have cited relevant literature to support the relevant content, see page 3, line 36 for specific changes.

We have followed your suggestions by providing paragraph summaries and research hypotheses in each subsection of the Theoretical Analysis and Research Hypotheses section. See pages 8-11 for specific changes.

**Commentator's Opinion:**

**In "Research Design," the number of companies analyzed should be better specified. It is suggested to first, describe the variables used "RDit," "Patentit," and "ESGit," arguing concisely why they are the ones used and, useful for the research. Personal curiosity: why are these variables the best compared to all other potential; variables available?**

**Author's Response:**

We agree with your suggestions.

Firstly, in the ‘Research design’, the article states the number of companies analysed, see line 242.

For firms‘ innovation investment measured by R&D investment, this paper refers to Shen Yi's study (https://doi:10.19616/j.cnki.bmj.2016.02.007.) The absolute value of R&D investment indicator, i.e., calculated as the natural logarithm of the amount of R&D investment can portray firms’ R&D behaviours in a more complete way. See line 280 for specific changes.

For the enterprise green innovation output measured by the number of green patents, firstly, the patent data can reflect a more specific output of the invention process, in addition, combined with China's information environment, the patent application indicator is more reflective of the enterprise's innovation research data. Therefore, this study refers to related research to measure green innovation output by the number of green patent applications of enterprises (https://doi.org/10.1016/j.iref.2024.103461). See line 285 for specific modifications.

For the measurement of ESG performance ability, we refer to related research methods (<https://doi.org/10.1371/journal.pone.0301701>;

https://doi.org/10.1371/journal.pone.0310447), and in addition, we combine China's national conditions and ESG-related data collection limitations to reflect the ESG performance of the sample companies. collection constraints, this paper adopts CSI ESG ratings to reflect the ESG performance of the sample companies. For details, please refer to line 292.

**Commentator's Opinion:**

**Other comments include implications, data and conclusions.**

**Why should this horizon be of interest to the reader? Moreover, could the pandemic of COVID invalidate his analysis?**

**I suggest that the authors better specify, in a dedicated section, the current limits of the research and possible future developments, as I believe this topic is highly topical.**

**Author's Response:**

We agree with your suggestions. Our main research direction is pharmaceutical enterprise innovation, combined with the current Chinese government's emphasis on the sustainable transformation of China's manufacturing enterprises, we believe that pharmaceutical enterprises, as high-tech manufacturing enterprises, are one of the most important subjects to carry out the transformation of the sustainable conversation, and therefore choose to explore whether the active disclosure of ESG performance by enterprises in the process of the sustainable transformation of pharmaceutical enterprises will have a positive impact on their innovation inputs and outputs. impact on their innovation input and output.

We added a Limitations and Perspectives section to the Conclusions and Recommendations section for a description of the current study, based on the reviewer's suggestions about the impact of the new crown epidemic on the analyses and the timeliness of the topic. See line 623 for specific changes.
