# Supplementary material for: The impact of ESG performance on corporate innovation—Empirical evidence from Chinese pharmaceutical listed companies
Source: PLoS One. 2025 Nov 4;20(11):e0332906. doi: 10.1371/journal.pone.0332906 (PMC12585053; doi:10.1371/journal.pone.0332906)
Supplement: Attachment — Submitted filename: Response_to_Reviewers_auresp_2.docx [file pone.0332906.s005.docx]

**Response to Reviewers**

We are very grateful to the reviewers for their review and valuable comments on our papers. Your suggestions are crucial to improve the quality of the paper. We have made changes according to your comments and our response is as follows:

**Commentator's Opinion:**

**This paper has some concerns regarding the rationale for selecting only pharmaceutical listed companies, requiring a detailed discussion on potential biases and their impact. Separating the "Institutional Background" into its own paragraph and providing a deeper explanation of the industry's suitability would enhance clarity and credibility.**

**Author's Response:**

We agree with you on these suggestions. The relevant paragraphs of the introduction have been reorganized, and since the relationship between sustainability and corporate innovation is one of the key concerns of pharmaceutical companies, the main research target of this paper is pharmaceutical companies. Firstly, an explanation and discussion of the applicability of ESG performance to the pharmaceutical industry has been added to the introduction section, which is believed to be better in the revised version. See page 4 for specific modifications.

In addition, some of the article's limitations have been reorganized at the end of the article, with the main additions being the opportunity to further explore the differences in the impact of ESG performance on corporate innovation in pharmaceutical segments (e.g., biopharmaceuticals and traditional Chinese medicines, etc.) in the future, or to introduce a comparative study of other industries with the pharmaceutical industry. See page 45 for specific changes.

**Commentator's Opinion:**

**While revising your submission, please upload your figure files to the Preflight Analysis and Conversion Engine (PACE) digital diagnostic tool. PACE helps ensure that figures meet PLOS requirements.**

**Author's Response:**

We agree with your suggestion. We uploaded Figure 1 to PACE, and completed the modification according to the requirements, and the modified image meets the requirements of PLOS.
