# Supplementary material for: The impact of ESG performance on corporate innovation—Empirical evidence from Chinese pharmaceutical listed companies
Source: PLoS One. 2025 Nov 4;20(11):e0332906. doi: 10.1371/journal.pone.0332906 (PMC12585053; doi:10.1371/journal.pone.0332906)
Supplement: Attachment — Submitted filename: review.docx [file pone.0332906.s004.docx]

**Peer Review of Manuscript: "The Impact of ESG Performance on Corporate Innovation – Empirical Evidence Based on Chinese Pharmaceutical Listed Companies"**

Recommendation: Recommended for publication with some minor Revision

1. General Comments:

This manuscript examines the impact of Environmental, Social, and Governance (ESG) performance on corporate innovation among A-share listed pharmaceutical firms in China, using panel data from 2015 to 2022. It employs fixed effects and mediation models to explore the direct and indirect effects of ESG, incorporating heterogeneity analysis and robustness checks. The paper addresses a relevant topic and is methodologically well-structured

2. Contribution to the Field:

The paper contributes meaningfully to the ESG and innovation literature by focusing on a critical and under-researched industry. The dual focus on both innovation input and green innovation output is a notable strength. The incorporation of mediators (R&D personnel, government subsidies) and heterogeneity analysis across firm characteristics offers depth to the empirical findings.

3. Specific Comments:

Title and Abstract:

- The title accurately reflects the content. However, consider rephrasing for smoother grammar: "Empirical Evidence from Chinese Pharmaceutical Listed Companies".
- The abstract clearly outlines objectives, methodology, key findings, and implications.

Introduction:

- The introduction is thorough and offers good context on ESG policy developments in China. However, the theoretical rationale is fragmented.

Methodology:

- The empirical strategy is sound. Fixed effects, mediation modeling, and instrumental variable (2SLS) methods are appropriate.
- Clearly explain the rationale behind using CSI ESG ratings and their scoring system. A table detailing ESG score distribution across years/firms could help.

Data and Variables:

- The innovation input (log R&D expenditure) and output (green patents) are standard but consider briefly addressing limitations of patent counts (e.g., time lags, quality variation).
- The variable descriptions are useful but could be more reader-friendly if organized in a summary table with examples.

Results and Discussion:

- The results are extensive and well-reported.
- The robustness checks (lagged ESG, quantile regression, Poisson, GMM) are impressive.
- The discussion of heterogeneity by ownership, region, and size is valuable.

Language and Style:

- The manuscript has many grammatical and stylistic issues (e.g., awkward phrasing, inconsistent tense, punctuation). A professional English editing service is recommended.
- Tables are dense and occasionally unclear. Consider splitting long tables or highlighting key coefficients.

Conclusion and Policy Implications:

- Conclusions are justified by the results.

4. Final Evaluation:

This is a promising paper with empirical rigor and relevance. Accepted with minor revisions as noted above will substantially improve the manuscript's clarity and theoretical depth, making it a valuable contribution to ESG and innovation studies.
