# Supplementary material for: The impact of ESG performance on corporate innovation—Empirical evidence from Chinese pharmaceutical listed companies
Source: PLoS One. 2025 Nov 4;20(11):e0332906. doi: 10.1371/journal.pone.0332906 (PMC12585053; doi:10.1371/journal.pone.0332906)
Supplement: Attachment — Submitted filename: Response_to_Reviewers_auresp_3.docx [file pone.0332906.s006.docx]

**Response to Reviewers**

We are very grateful to the reviewers for their review and valuable comments on our papers. Your suggestions are crucial to improve the quality of the paper. We have made changes according to your comments and our response is as follows:

**Commentator's Opinion:**

**Title and Abstract: The title accurately reflects the content. However, consider rephrasing for smoother grammar: "Empirical Evidence from Chinese Pharmaceutical Listed Companies".**

**Author's Response:**

We agree with you on these suggestions. We have modified the title in accordance with the suggested changes.See page 1 for specific modifications.

**Commentator's Opinion:**

**Introduction: The introduction is thorough and offers good context on ESG policy developments in China. However, the theoretical rationale is fragmented.**

**Author's Response:**

We agree with your suggestion.We considered that placing the theoretical basis in the introduction section would make that section too lengthy, so we moved the content related to the theoretical basis of ESG and corporate innovation to the second section, ‘Theoretical basis and research hypothesis.’。See page 6-7 for specific modifications.

**Commentator's Opinion:**

**Methodology:Clearly explain the rationale behind using CSI ESG ratings and their scoring system. A table detailing ESG score distribution across years/firms could help.**

**Author's Response:**

We agree with you on these suggestions.We added descriptive statistics for the ESG scores of the research samples from 2015 to 2022 and conducted a descriptive analysis of the statistical results.See page 18-20 for specific modifications.

In addition, descriptive statistics tables and DO files for corporate ESG scores from 2015 to 2022 have been re-uploaded to the data management platform to improve the reproducibility of the results. The specific path is as follows:

[*https://data.mendeley.com/preview/4r554m7kjm?a=e55a37e4-5626-491d-bd2b-a7558a382ce0*](https://data.mendeley.com/preview/4r554m7kjm?a=e55a37e4-5626-491d-bd2b-a7558a382ce0)

**Commentator's Opinion:**

**Data and Variables:**

**The innovation input (log R&D expenditure) and output (green patents) are standard but consider briefly addressing limitations of patent counts (e.g., time lags, quality variation).**

**The variable descriptions are useful but could be more reader-friendly if organized in a summary table with examples.**

**Author's Response:**

We agree with your suggestion.

First, we conducted an in-depth consideration and relevant tests on the issue of patent count restrictions in the robustness test section.On the one hand, considering the time lag effect of corporate ESG performance on corporate innovation, this study uses the lagged value of the explanatory variable for regression analysis. On the other hand, considering that the number of patent applications is a count variable, using only a fixed-effects model for regression analysis may not be robust enough. Therefore, this study re-conducts empirical tests using a Poisson regression model to verify the correlation between the main research variables.See page 24-26 for specific modifications.

Second, we summarized all variables in tabular form, as shown in Table 1.See page 16-18 for specific modifications.

**Commentator's Opinion:**

**Language and Style:**

**The manuscript has many grammatical and stylistic issues (e.g., awkward phrasing, inconsistent tense, punctuation). A professional English editing service is recommended.**

**Tables are dense and occasionally unclear. Consider splitting long tables or highlighting key coefficients.**

**Author's Response:**

We agree with your suggestion.

Firstly, we have thoroughly reviewed and revised the wording and tense throughout the entire text.

Secondly, to address the issue of excessive tables, we have removed the presentation of control variable data and uploaded all complete tables as Supporting Information.

Additionally, we have added a legend to Figure 1 to facilitate readers' understanding of the image's content.
